# Sex-stratified insights into the genetics of brain volumes in late adulthood

**DOI:** 10.1186/s13293-026-00943-4

**Published:** 2026-07-14

**Authors:** Édith Breton, Elleke Tissink, Gloria Matte Bon, Dennis van der Meer, Ole A. Andreassen, Tobias Kaufmann

**Affiliations:** 1 Centre for Precision Psychiatry, Division of Mental Health and Addiction, Institute of Clinical Medicine, University of Oslo, Oslo, Norway; 2 Centre intersectoriel en santé durable, Département des sciences fondamentales, Université du Québec à Chicoutimi, 555, Bd de l’Université, QC G7H 2B1 Saguenay, Canada; 3 Eating Disorders Continuum & Douglas Research Centre, Douglas Mental Health University Institute, Montreal, Canada; 4 Department of Psychiatry and Psychotherapy, Tübingen Center for Mental Health, University of Tübingen, Calwerstr. 14, 72076 Tübingen, Germany; 5 Department of Women’s and Children’s Health, Science for Life Laboratory, Uppsala University, Uppsala, Sweden; 6 German Center for Mental Health (DZPG), Partner site Tübingen, Tübingen, Germany

**Keywords:** Sex differences, Brain anatomy, Imaging genetics

## Abstract

**Background:**

The prevalence, timing and disease course of mental and neurological disorders vary according to sex, yet the neurogenetic mechanisms underlying sex differences in brain disorders remain poorly understood. While sex chromosomes and hormones contribute to sex differences in brain biology, previous studies suggest a role for autosomal genetic variation as well.

**Methods:**

We investigated autosomal genetic associations with brain volumes in 15,740 females and 15,740 males from the UK Biobank, matched for age and scan site, using sex-stratified genome-wide association analyses. We applied a multivariate genome-wide approach (MOSTest) across 257 brain regions and complemented these analyses with region-specific univariate genome-wide association studies.

**Results:**

Heritability estimates and genetic correlations were highly similar between females and males in late adulthood, indicating largely shared genetic influences on brain volumes. Many loci reaching genome-wide significance in one sex also showed signal in the other. Gene mapping in these loci yielded a greater total number of brain-volumes associated genes in females than in males. Variability in the number of mapped genes was particularly pronounced in limbic regions such as the insula, cingulate cortex, hippocampus and amygdala.

**Conclusion:**

Overall, our findings contribute to a better understanding of autosomal genetic influences on brain volumes in males and females and may inform future studies examining sex variability in neurobiological mechanisms relevant to brain disorders.

**Supplementary Information:**

The online version contains supplementary material available at https://doi.org/10.1186/s13293-026-00943-4.

## Abstract (plain language summary)

Mental and neurobiological disorders often differ between females and males. Some disorders are more common in one sex than the other, and symptoms can present or evolve differently. Variation in brain biology, partly shaped by genetic factors, may contribute to these patterns. In this study, we investigated genetic factors linked to brain structure in females and males separately. We focused on the volumes of 257 brain regions and analyzed genetic data from more than 30,000 adults from the UK Biobank. Overall, we found that the genetic influences on brain volumes are largely similar between females and males in late adulthood. At the same time, we observed that the number of genes associated with brain volumes was higher in females than in males. These variations in number of genes were marked in brain regions involved in the limbic system. Together, our findings improve our understanding of how genetics contribute to brain structure in females and males and may inform future studies examining sex-related variation in brain disorders.

**Supplementary Information:**

The online version contains supplementary material available at https://doi.org/10.1186/s13293-026-00943-4.

## Highlights

Genome-wide influences on brain volumes are highly correlated between females and males in late adulthood.

Gene-level variability was observed, with a higher number of genes mapped from genome-wide significant loci in females than in males in both multivariate and univariate analyses.

This gene-level variability was most pronounced in limbic regions, such as the insular and cingulate cortices.

**Supplementary Information:**

The online version contains supplementary material available at https://doi.org/10.1186/s13293-026-00943-4.

## Background

Sex impacts the prevalence, clinical presentation, course and treatment response of several mental and neurological disorders [1, 2]. Major depressive disorder and Alzheimer’s disease are twice as common in females as in males, and anorexia nervosa is 10 times more common in females than in males [3–5]. In contrast, autism spectrum disorder, alcohol use disorder and attention deficit hyperactivity disorder are two to three times more common in males [6–8]. The main windows of vulnerability for mental disorders also differ by sex, with females more likely to be affected by conditions that begin in adolescence or later, and males more likely to develop neurodevelopmental disorders that manifest in childhood. Although neuroimaging studies have identified structural and functional differences between male and female brains across the lifespan [9], the biological mechanisms underlying sex variability in brain health remain incompletely understood [10–12]. Such mechanistic understanding could facilitate efforts toward precision medicine in the clinical neurosciences and contribute to reducing sex inequalities in health care and biomedical research [13, 14].

Sex chromosomes and hormones are well-established contributors to sex differences in the brain, but they are not the only ones [15, 16]. Increasing evidence suggests that autosomal genetic variations also play a role in shaping brain-related traits and disorders in a sex-dependent manner [17–21]. For example, genetic studies have identified sex-dependent associations between autosomal variants and mood or psychotic disorders [17], and interactions between autosomal variants and sex hormones have been proposed to influence the risk of dementia [19]. In addition, autosomal genetic variants can influence gene expression in a sex-dependent manner across multiple human tissues, including the brain [18, 20, 21]. For example, sex differences in transcriptional regulation have been reported for genes involved in neurotransmission, immune processes and psychiatric disorders [20, 22, 23]. Throughout the life course, genetic factors may further interact with biological processes such as neurodevelopment, ageing, inflammation, and epigenetic regulation to shape brain structure differently in males and females [24–26]. Together, these findings suggest that genetic variants may operate differently in the brains of females and males. However, the extent to which such genetic variants are linked to brain structure in males and females remains poorly understood. To date, most imaging genetics studies have analyzed males and females jointly, potentially overlooking sex-dependent patterns of genetic associations that may be relevant for brain health and disorders [14].

Here, we investigated the genetic underpinnings of brain volumes in 15,740 females and 15,740 males from the UK Biobank. Both groups were matched for age and scan site. Our overarching aim was to describe autosomal genetic associations with brain volumes in females and males separately. We opted for sex-stratified analyses to determine whether the overall patterns of autosomal genetic associations observed in one sex were also evident in the other. To this end, we extracted volumes of 180 cortical and 77 subcortical regions across the brain for genome-wide association studies (GWAS). We leveraged MOSTest [27], a multivariate GWAS tool designed to enhance power and facilitate discovery in imaging genetics research, yielding one multivariate summary statistic across the whole brain for females and males separately. In addition, we assessed each brain region in females and males separately using a univariate approach.

## Methods

### Sample

We selected participants from the UK Biobank with genetic and neuroimaging data and of European descent, defined by self-identification as “White British” and similar genetic ancestry based on genetic principal components analysis (accession code no. 27412). Study procedures were approved by local institutional review boards, and each sample was collected with participants’ written informed consent. Standardized brain magnetic resonance imaging (MRI) and genomic protocols are described elsewhere [28–30].

### Magnetic resonance imaging

The present study used T1-weighted MRI data obtained from UK Biobank, which were processed using FreeSurfer (https://surfer.nmr.mgh.harvard.edu/). Cortical regions of interest (ROIs) were defined using the Glasser Atlas, a multimodal cortical parcellation derived from the Human Connectome Project [31]. This high-resolution atlas allows structural variation to be characterized across 180 cortical ROIs per hemisphere, making it well suited to investigate distributed patterns of genetic associations across the brain. In addition, subcortical structures were obtained using standard FreeSurfer segmentation (10 ROIs [32]), together with further segmentation of the amygdala (9 nuclei + whole amygdala [33]), the hippocampus (19 subregions + whole hippocampus [34]), the thalamus (25 subregions + whole thalamus [35]), the brainstem (4 subregions + whole brainstem [36]) and the hypothalamus (5 subregions + whole hypothalamus [37]). In total, we considered 257 ROIs across the whole brain, using the mean of the right and left hemisphere volumes.

For each participant, the mean Euler number across the left and right hemispheres was computed. Participants whose mean Euler number fell more than three standard deviations below the sample mean were excluded from the analyses. This left us with 34,706 participants of European descent with both genetics and neuroimaging data. From these 34,706 participants, we selected male-female pairs matched on scan site and age (within a 6-months difference). Among potential matches, pairs were selected to minimize differences in estimated total intracranial volume, following a matching strategy previously used by others [38]. The goal of this procedure was to reduce large global differences in brain size between sexes while maintaining comparable demographic and imaging characteristics. Participants who did not meet matching criteria were excluded, leading to the inclusion of 15,740 males and 15,740 females with a mean age of 64,4 years.

### Genome-wide analyses

We deployed the Multivariate Omnibus Statistical Test (MOSTest) [27] to perform two multivariate GWAS of the whole brain volume, in females and males separately, focusing on autosomes. As in previous studies using MOSTest, individuals with genotype missing rate > 10%, variants with genotype missing rate > 5%, variants failing Hardy-Weinberg equilibrium at *p*=1 × 10^− 9^, variants with minor allele frequency below 0.5%, and imputation info score below 0.5 were filtered out [39–41]. We controlled our analyses for covariates by pre-residualizing the ROI volumes for age, age^2^, scanning site, the first 20 genetic principal components (derived from UK Biobank [28]), mean hemispheric Euler number, and estimated total intracranial volume (in R version 4.2) prior to GWAS in females and males separately. Estimated total intracranial volume was included to account for global differences in head size across individuals. These covariates were selected based on previous imaging genetics studies conducted in the UK Biobank to account for demographic, technical and population structure confounders [41, 42].

In parallel, we extracted univariate GWASs from the univariate stream of MOSTest [27] for each of the 257 ROIs, to gain a better understanding of the regional heterogeneity that may occur between males and females in specific brain regions.

### Distribution of association signals across sex

To assess how association signals within loci identified in one sex were distributed in the other, we visualized *p*-values across sexes. First, for each locus identified in the multivariate GWAS for one sex, we identified the top lead SNP (with the smallest p-value). Then, we searched within the same locus boundaries (based on based on chromosomes, loci start positions and end positions as defined by FUMA; see below) in the multivariate GWAS results of the other sex and identified the SNP with the smallest *p*-value within that locus. The -log10(*p*-value) of these minimum *p*-values were then used to visualize how association signals observed in one sex were distributed in the other. The same procedure was applied for loci identified in both females and males.

### FUMA

The Functional Mapping and Annotation of Genome-Wide Association Studies (FUMA) platform [43] v1.5.2 was used to define genome-wide significant loci (*p* < 5E-8) and perform positional gene mapping for all candidate SNPs, using the default settings of the SNP2GENE functionality. Thus, the number of genes reported in the Results section correspond to the number of genes mapped from SNPs in loci significantly associated with brain volumes, using FUMA, and multiple genes can map to the same locus. For the list of overlapping genes between females and males, as well as the genes mapped to loci reaching genome-wide significance only in females or only in males based on multivariate analyses, FUMA GENE2FUNC was used to perform enrichment analyses against 15,496 gene sets (curated gene sets: 5,500; Gene Ontology terms: 9, 996) from MsigDB version v7.0. Tissue enrichment analyses were also performed using gene expression data from the GTEx project (v8) implemented in FUMA. We corrected for multiple testing using FDR and set the minimum overlapping genes to 2.

### Heritability & genetic correlations

The sex-stratified univariate GWASs summary statistics were used to compute heritability and genetic correlations estimates for each ROI between females and males, using linkage disequilibrium score regression (LDSC) [44, 45] with default settings and LD Scores based on the European 1000 Genomes reference panel. To reduce noise and improve statistical efficiency [46, 47], phenotypes with low heritability z-scores (*h*^2^_SNP_ / SE < 1.96) were excluded from further analyses because their SNP-based heritability was not significantly different from zero.

## Results

### Multivariate genetic associations and functional enrichments show substantial overlap across sexes alongside a higher mapped gene count in females

**Fig. 1:**
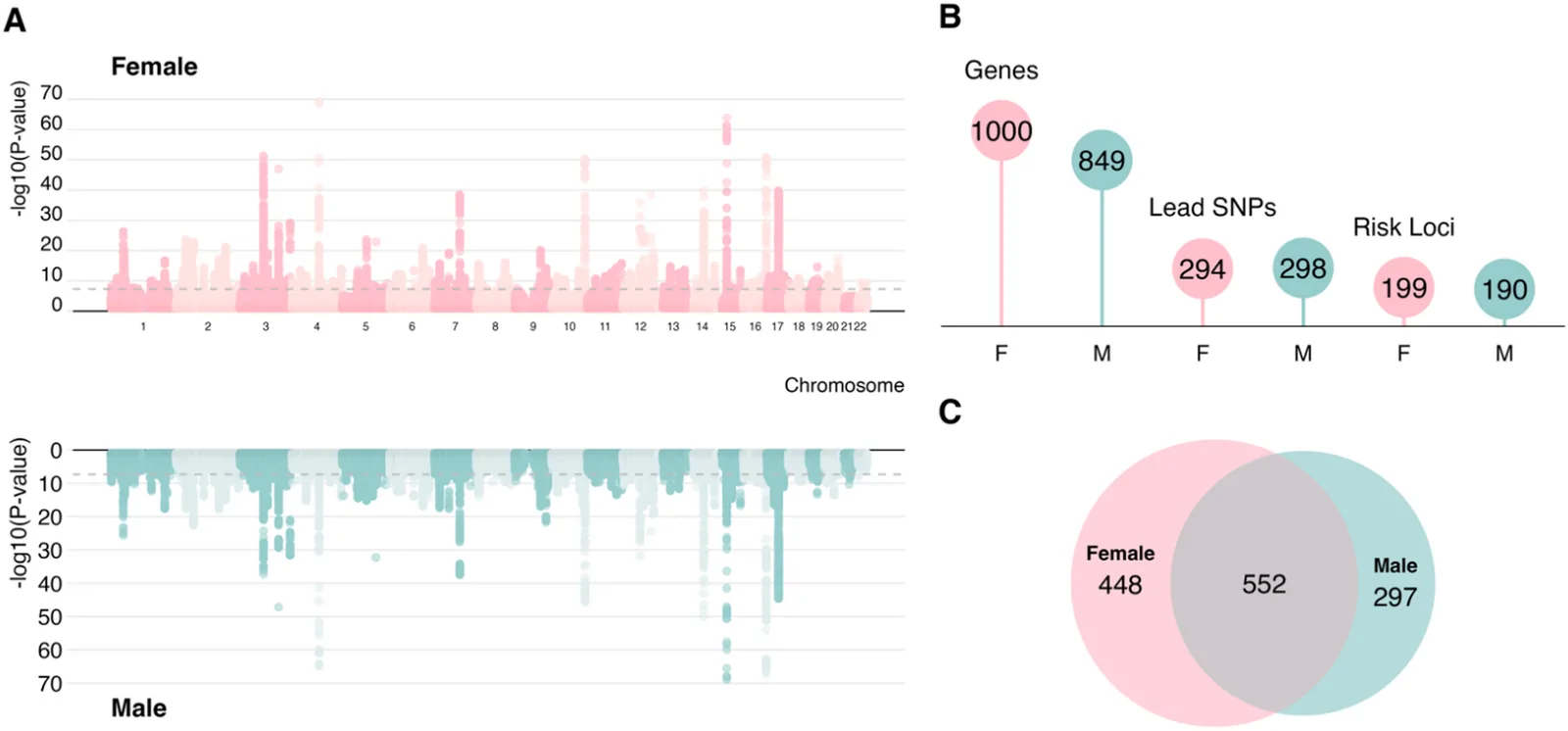
(**A**) Miami plot displaying the multivariate genome-wide association statistics in females (top/pink) and males (bottom/blue). These statistics reflect the association across 257 brain regions of interest. (**B**) Comparison of the number of genes, lead single nucleotide polymorphisms (SNPs) and risk loci identified in females (pink) versus males (blue) based on the multivariate GWAS statistics. The number of risk loci correspond to the number of loci with at least one gene mapped. (**C**) Venn diagram showing the corresponding number of genes overlapping or not in females (pink) and males (blue)

**Fig. 2:**
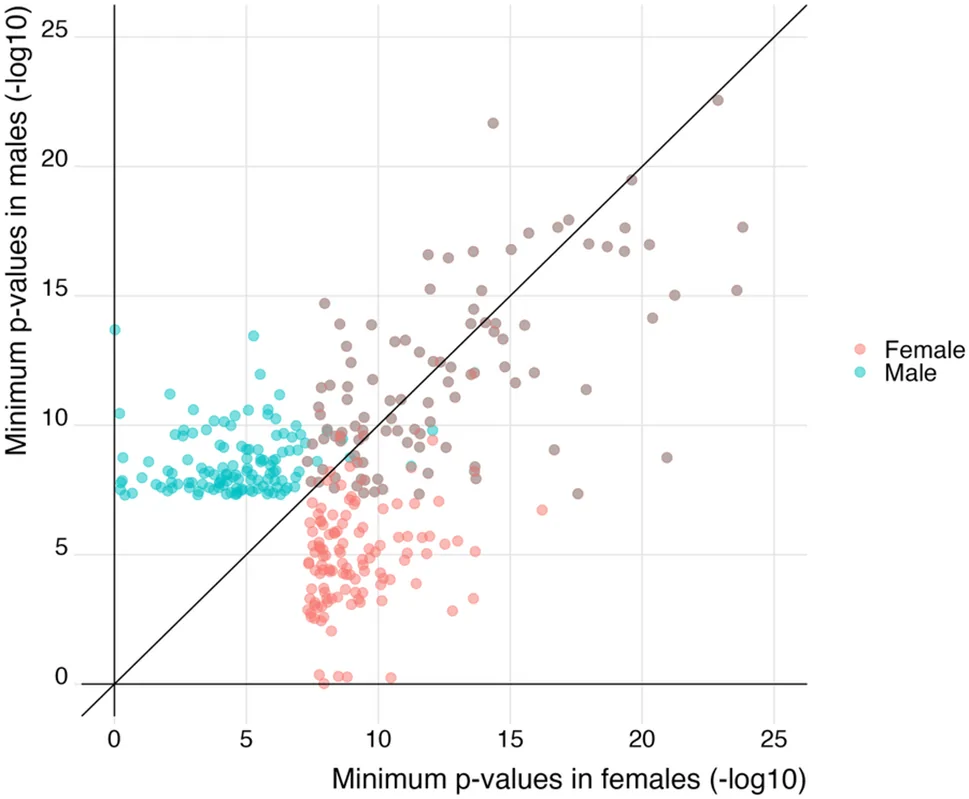
Distribution of association signals across sexes for all loci identified in the sex-stratified multivariate GWASs (i.e., in males and/or in females). For each locus identified in one sex, the lead SNP (lowest p-value) was identified and the smallest p-value within the same locus was extracted from the multivariate GWAS results of the other sex

Using a multivariate GWAS framework, we identified 231 genome-wide significant loci (*p* < 5E-8) associated with brain volumes in females and 241 loci associated with brain volumes in males, including 127 physically overlapping loci between both sexes (based on chromosomes, loci start positions and end positions). Genome-wide patterns of association appeared highly similar between sexes (Fig. 1A). Visual inspection of association signals across sexes indicated that many loci identified in one sex also showed *p*-values> 5E-8 in the other sex, although the strength of association varied, and some loci showed little or no signal in the other sex (*p*-values > 0.05) **(**Fig. 2**)**. Genetic variants in 199 (female) and 190 (male) loci could positionally be mapped to genes, leading to the identification of 1000 genes associated with brain volumes in females and 849 genes associated with brain volumes in males (since in some loci, many genes were mapped; Fig. 1B). Approximately half of the identified genes (*N* = 552) overlapped between males and females, while 448 genes were identified only in females and 297 genes were identified only in males at the genome-wide significance threshold (*p* < 5E-8; Fig. 1C, Supplementary Table 1).

To provide biological context for the genes identified in the multivariate analyses, we performed functional annotation using FUMA (Supplementary Figs. 1–6). Genes shared between females and males were significantly (*p*_*FDR*_ < 0.05) enriched in 123 gene-sets (Supplementary Fig. 1). Twenty-five of these gene-sets were related to neurodevelopmental pathways (e.g., neuron development, neurogenesis, regulation of neuron projection), 15 were related to developmental/growth processes, and 15 were related to immune pathways. Tissue enrichment analyses further showed that genes shared between males and females were differentially expressed (*p*_*FDR*_ < 0.05) in 21 tissue types. These included 11 key brain regions such as the putamen, nucleus accumbens, caudate nucleus, amygdala, anterior cingulate cortex, hypothalamus and hippocampus, frontal cortex, and substantia nigra (Supplementary Fig. 2).

Among the 448 genes mapped from loci reaching genome-wide significance only in female brain volumes, enrichment analyses identified 23 tissue-specific gene-sets with differential expression, of which 13 were brain tissues (Supplementary Fig. 3). Most of these (*N* = 19) overlapped with the enrichment pattern of genes shared between females and males described above, except for cerebellum, cerebellar hemisphere, tibial artery and EBV-transformed lymphocytes. Genes only significant in females also showed enrichment in biological processes related to ion transport, cellular homeostasis, and phosphorylation (*N* = 13 gene-sets; Supplementary Fig. 4). 23% (*N* = 3) of these biological processes overlapped with the enrichment patterns of genes shared between females and males.

Among the 297 genes mapped from loci reaching genome-wide significance only in males, enrichment analyses identified 34 tissue-specific gene-sets with differential expression. These included 12 brain tissues that were also identified in genes only found for females or genes shared between males and females (Supplementary Fig. 5). Analyses indicated enrichment for biological processes related to neurodevelopment, neuronal morphogenesis, and central nervous system development (*N* = 40 gene-sets, Supplementary Fig. 6). 40% (*N* = 16) of these biological processes overlapped with the enrichment patterns of genes shared between females and males.

### Univariate cortical ROI analyses reveal high genetic correlations between the sexes with mapped gene count divergence in the insular and cingulate cortices

**Fig. 3:**
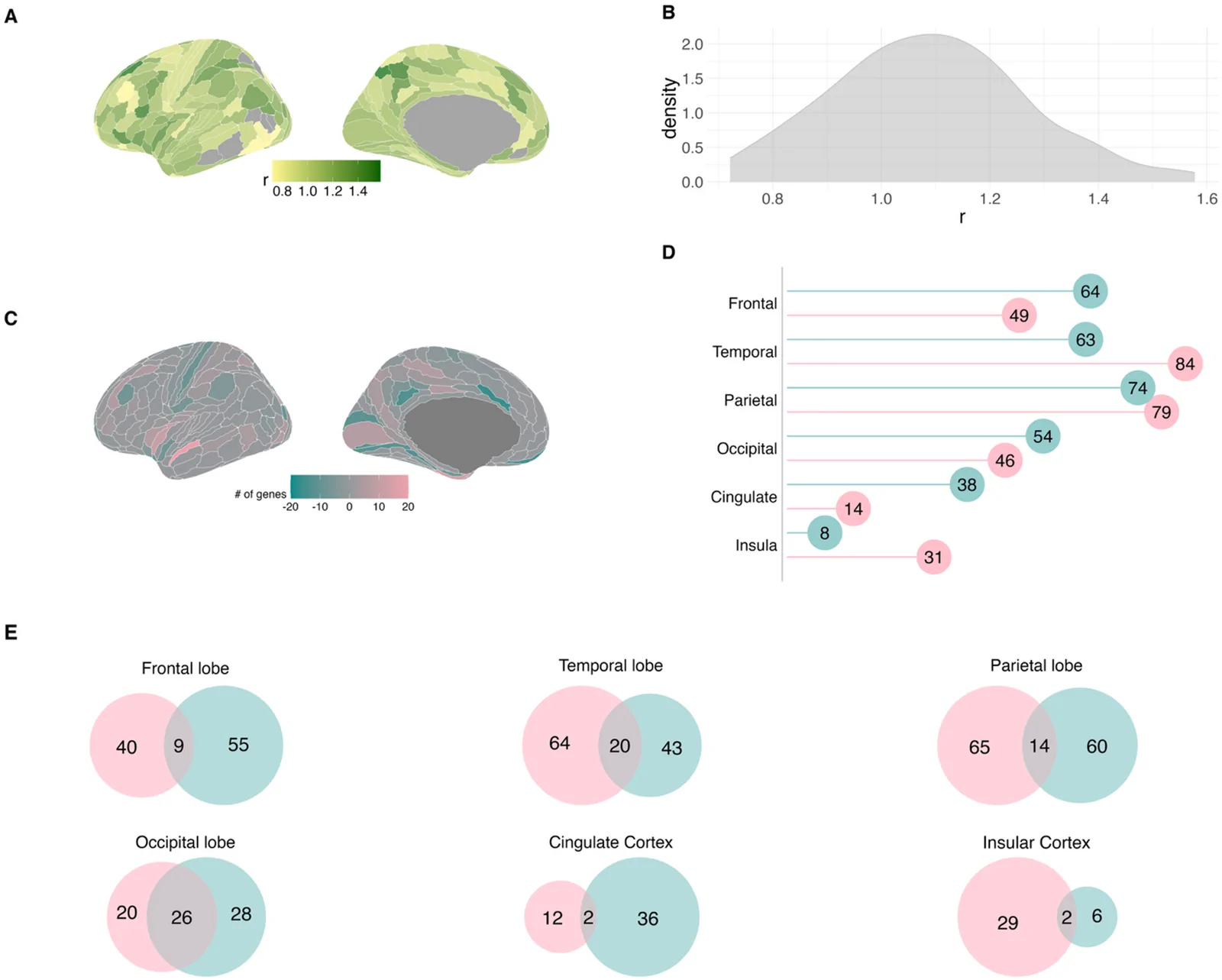
(**A**) Genetic correlations for each cortical ROI between males and females. Note that LDSC correlation estimates are not bound to 1 and can therefore exceed 1 in cases of high GWAS similarity and depending on the estimation error. (**B**) Density plot showing the distribution of genetic correlations between males and females for all cortical ROIs. (**C**) Differences between females and males (i.e., females – males) in the number of genes identified through univariate GWASs, for each cortical regions of interest. Pink shades indicate a higher number of genes in females, whereas blue shades indicate a higher number of genes in males. (**D**) Number of genes identified in females (pink) versus males (blue) in the 180 univariate GWASs for cortical ROIs, presented by brain lobe. (**E**) Venn diagrams showing the overlap of genes identified in females (pink) and males (blue) across brain lobes, based on univariate GWASs. Full gene lists can be found in S2-S7

**Fig. 4:**
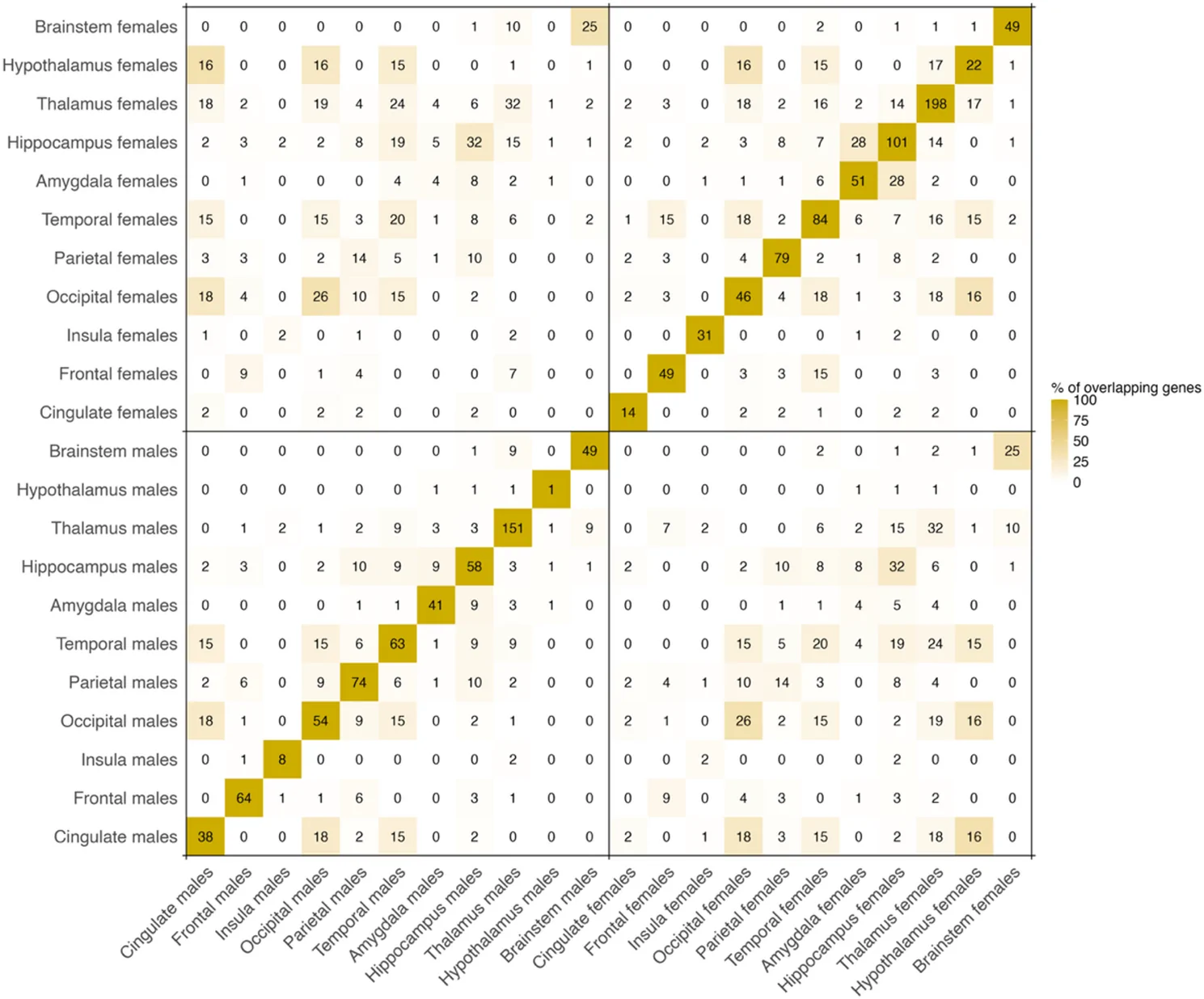
Heatmap showing the percentage of overlapping genes out of the total number of genes identified for two given regions, for all cortical and subcortical brain regions, and within or between sexes. Color scale represents the percentage of overlapping genes between two regions whereas the absolute number of overlapping gene is displayed within each cell. The figure is based on univariate GWAS results. Full gene lists can be found in Supplementary Tables 2–12

Next, we sought to characterize patterns of sex-stratified associations across distinct cortical brain regions. To do this, we turned to a univariate framework and performed standard GWASs for each of the 257 cortical regions of interest (ROI) separately. We further assigned cortical ROIs to their respective lobes (i.e., frontal, parietal, temporal, occipital, insula, cingulate). We performed sex-stratified LDSC [44, 45] to estimate SNP-based heritability across the 257 brain regions in females and males, yielding highly similar mean heritability estimates of *h*^2^ = 0.19 ± 0.07 in females and *h*^2^ = 0.18 ± 0.06 in males (Supplementary Fig. 6). Similarly, genetic correlations between sexes were consistently high in cortical and subcortical brain regions (based on LDSC; all *r* > 0.72; Figs. 3A-B and 4), indicating largely shared genetic influences on brain volumes. Despite this overall similarity, the number of genes mapped from loci reaching genome-wide significance differed between sexes across brain regions (Fig. 3C). Of the 257 ROIs examined, 198 had at least one gene mapped to a locus that reached genome-wide significance in at least one sex. Among these, only 17 ROIs (8.6%) showed a difference in gene counts between sexes of less than 10%. When combining all the unique genes identified in the univariate analyses across cortical and subcortical ROIs, we found that 171 genes overlapped between males and females. Meanwhile, 502 genes were identified only in females, and 407 genes were identified only in males at the genome-wide significance threshold (*p* < 5E-8). These univariate analyses revealed that differences in gene counts were distributed across a broad set of cortical and subcortical ROIs, suggesting that these differences in gene counts were not confined to a small subset of specific ROIs.

Next, we examined sex-dependent patterns at the lobe level (Fig. 3D**)**. Notably, the insular and cingulate cortices showed heterogeneity in the number of genes reaching genome-wide significance (*p* < 5E-8). The cingulate cortex was associated with twice as many genes in males versus females, with 38 genes identified compared to 14 in females, with only 2 genes overlapping (Fig. 3E**)**. In contrast, the insula was significantly associated with almost four times more genes in females (31 genes) than in males (8 genes), with only two genes overlapping between the sexes (Fig. 3E).

In addition, we examined the overlap of genes identified by univariate GWASs in the different brain regions, both within and between sexes. Our analysis revealed limited gene overlap across brain regions and within and between sexes, highlighting regional and potential sex-dependent variability at the gene level (Fig. 4).

### Divergent gene counts and limited gene overlap is most prominent in limbic structures

**Fig. 5:**
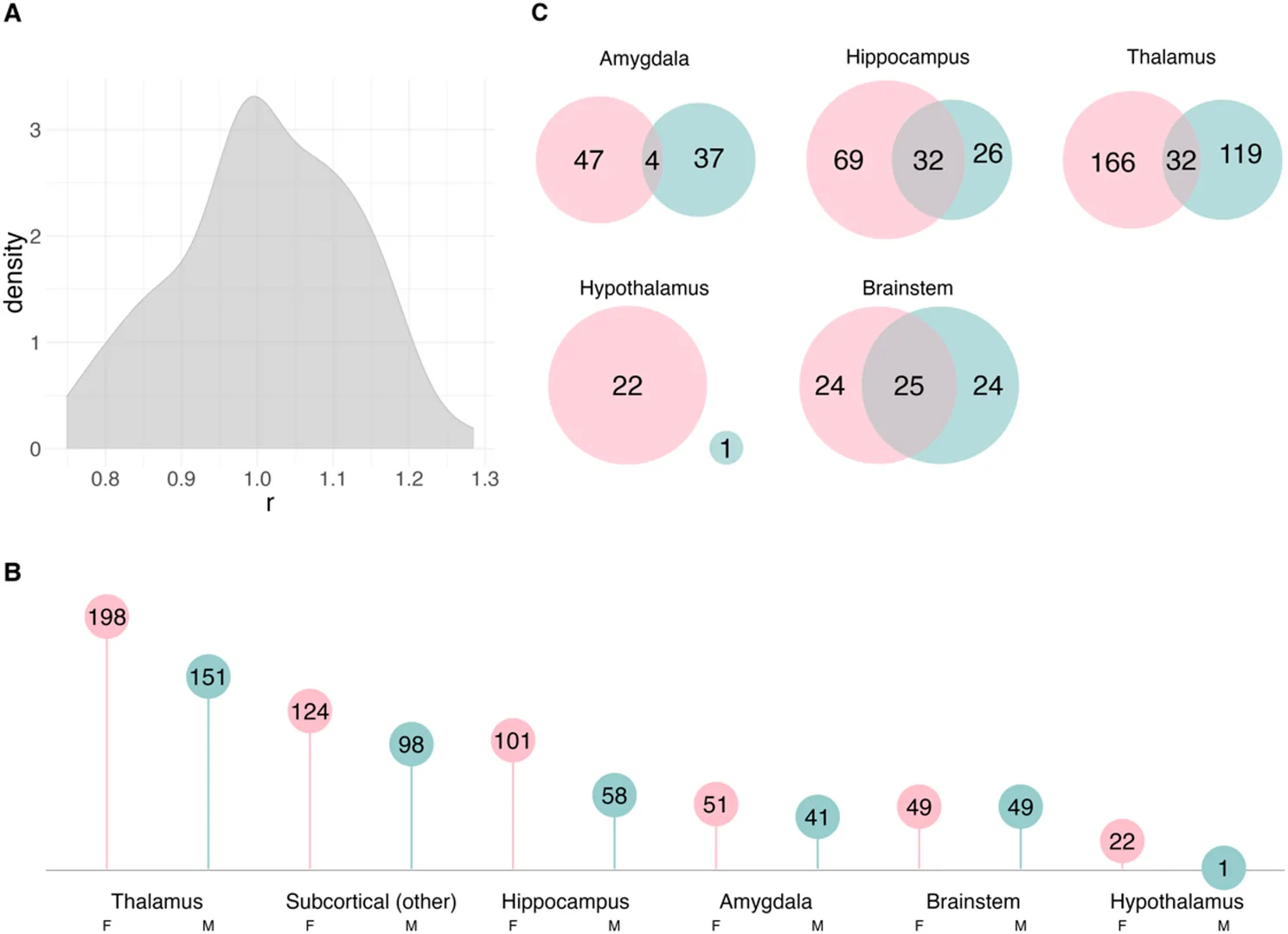
(**A**) Density plot showing the distribution of genetic correlations between males and females for all subcortical ROIs. (**B**) Comparison of the number of genes identified in females (pink) versus males (blue) by the 77 univariate GWASs for each subcortical ROI, presented by subcortical structure. (**C**) Venn Diagrams showing the number of genes identified by univariate GWASs in females (pink) and males (blue); for all ROIs located in subcortical structures. Full gene lists can be found in S8-S12

In line with the cortical results, univariate GWAS for subcortical volumes were also strongly correlated between males and females (Fig. 5A). All subcortical structures (except the brainstem) showed a higher number of mapped genes from loci reaching genome-wide significance (*p* < 5E-8) in females than in males (Fig. 5B).

The difference in mapped genes between females and males emerged most prominently within limbic structures such as the amygdala and the hippocampus (Fig. 5C; Supplementary Tables 8–9). In the hippocampus, we identified 69 genes mapped from loci only genome-wide significant in females and 26 genes mapped from loci only significant in males. In the amygdala, we found 47 genes in females, 37 genes in males and 4 overlapping genes. In the thalamus, 32 genes were mapped from loci significant in both GWAS, whereas we identified 166 genes that were mapped from loci reaching genome-wide significance only in females, and 119 genes that were mapped from loci reaching genome-wide significance only in males (Fig. 5C, Supplementary Table 10).

Lastly, there was no overlap between female and male genes for the hypothalamus (Fig. 5C, Supplementary Table 11).

## Discussion

The present study used a combination of multivariate and univariate genome-wide approaches to characterize patterns of genetic associations with brain volumes in males and females. Genetic correlations between the sexes were high across all brain regions, indicating largely shared genetic influences. Yet, we observed sets of genes mapped from loci that reached genome-wide significance in only one sex. Moreover, our results suggest that more genes are associated with brain volumes in females than in males overall, at least in late adulthood. This excess of genes mapped from genome-wide significant loci in females aligns with recent reports demonstrating a higher number of genomic loci with significant local heritability in females across a wide range of quantitative traits [48].

Particularly in limbic structures, we found different gene counts in females versus males, with many structures reaching a higher number of mapped genes in females compared to males. Responsible for emotions, learning and memory [49], the limbic system has been linked to variation between males and females, both in terms of brain structure and association with mental and neurological disorders. A previous study by our group found that limbic structures show greater volume differences between sexes than non-limbic structures, and that they are more heritable [50]. In the present study, the cingulate cortex was one of the few regions where more genes were identified in males compared to females. Sex differences in the cingulate cortex have been reported in healthy individuals [51], but also in people with autism spectrum disorders [52] and depression [53]. The cingulate cortex is also associated with impulsivity and aggressive behaviors in childhood, and these associations have been shown to differ between males and females [54, 55]. In line with this evidence, many genes only mapped from cingulate cortex associated loci in males have previously been associated with male-prevalent mental disorders, including autism spectrum disorders (e.g., *RPP25* [56]) and cocaine dependence (e.g., *NSF* [57]).

Enrichment analyses indicated that genes associated with brain volumes in females, males, or shared across sexes converged on biological processes related to neurodevelopment and neuronal function. Tissue enrichment analyses were consistent with these observations, as the female-only, male-only and overlapping gene sets were all enriched in multiple brain regions (e.g., basal ganglia, amygdala, cingulate cortex, hypothalamus, hippocampus). Together, these findings show that the genetic signals identified in females and males map onto brain-relevant biological processes. At the same time, some differences emerged between sex-dependent gene sets. Genes identified only in females were enriched in processes related to cellular regulation, including cellular transport and homeostasis, whereas genes identified only in males were commonly associated with central nervous system development and cell morphogenesis. The enrichment of genes related to cellular processes in the female gene-sets may be consistent with mechanisms involved in neuronal plasticity and cellular adaptation [58, 59]. The latter are known to be sensitive to hormonal fluctuations across the lifespan, including during major life transitions such as pregnancy or menopause, which have been associated with structural and functional changes in the brain as well as shifts in vulnerability to mental disorders [58–61]. In males, the enrichment of genes related to central nervous system development and cell morphogenesis may reflect processes involved in the structural organization of the brain early in life. This interpretation is consistent with literature highlighting sex differences in early brain development and in the prevalence of neurodevelopmental conditions [6, 7, 26].

Relatedly, females and males may experience various environmental exposures or life events (e.g., trauma, pregnancy, parenting), during which the brain undergoes adaptations that may have long-lasting effects [62, 63]. Genetic regulatory mechanisms may modulate these adaptations [64]. These include epigenetic modifications and other mechanisms that regulate gene expression – factors increasingly recognized as key players in sex-related variation in human tissues [22, 65, 66]. Consistent with this broader literature, several genes mapped from loci reaching genome-wide significance in only one sex in our analyses are involved in the regulation of gene expression, including transcriptional regulation (e.g., *FOXP2*, *TP53*, *RFX4*), chromatin organization and epigenetic processes (e.g. *BLTP3A*, *ASH1L*, *PHF21A*). Future work will be needed to elucidate how these regulatory mechanisms interact with environmental triggers and contribute to variability in brain structure and risk of brain disorders in females and males.

The present univariate analyses have also identified several genes mapped from genome-wide significant loci which were previously implicated in neurotransmission and neuropsychiatric traits. This includes *SLC6A4* (identified in the female insula), the molecular target of selective serotonin reuptake inhibitors (SSRIs), which has been associated with sex-dependent effects in depression [67], obsessive-compulsive symptoms [68], and bipolar disorder [69]. Another gene identified in females, in the insula, is *GRID2*, a glutamate receptor possibly associated with antipsychotic response [70] and previously associated with obsessive-compulsive symptoms in females but not in males [71]. In the hippocampus, *GRIN2A*, a component of the NMDA receptor complex previously associated with schizophrenia [72], bipolar disorder [73] and depression [74], reached genome-wide significance only in females in our analyses. In contrast, an example of neurotransmission-related gene mapped from a locus reaching genome-wide significance only in males is the GABA receptor *GABBR1*, previously associated with schizophrenia, bipolar disorder, depression and autism spectrum disorder [75], and associated here with male thalamic volume. In line with previous work highlighting interactions between sex hormones and neurotransmission pathways [76], these observations raise the hypothesis that differences in the functioning of neurotransmission-related pathways may contribute to variability in brain structure between females and males. Further work will be required to clarify this hypothesis.

We also identified genes associated with male and female brain volumes that may represent candidate loci for future genetic research examining links between brain structure and common brain disorders in females and males. As an example, in the hippocampus and thalamus of females—but not males—we respectively found *CARF*, *NBEAL1*, and *LRP11*, as well as *CRHR1*, *NSUN3*, and *NCKIPSD*. These genes have previously been associated with neuropsychiatric traits, including eating disorders and anxiety, conditions that exhibit sex differences in prevalence and clinical presentation [77, 78]. In contrast, the genes that we identified only in males were frequently involved in male-prevalent conditions that typically onset in childhood, such as autism spectrum disorders, intellectual disability and epilepsy (e.g., *AUTS2* [79], *RPP25* [56], *SLC1A2* [80]). Overall, these findings provide candidate loci that could inform future research into the relationship between genetic influences on brain structure and sex-related variability in brain disorders. A better understanding of these neurogenetic mechanisms may eventually help clarify the behavioral and clinical sex differences observed across neuropsychiatric conditions [1, 10, 81, 82].

While our results provide important new insights into sex patterns in neurogenetics, they need to be interpreted in the light of some limitations. We studied a population of adults aged 40–80, and the link between genetics, brain and disorder risks may not be the same at all stages of life. Although we minimized the impact of age on our results by carefully matching males and females on age, it will be interesting to explore, in the future, the degree of stability in the observed associations across the lifespan. Another limitation of this study is that the analyses were restricted to individuals of European ancestry. Future large-scale imaging genetics studies including more diverse ancestry groups will be important to evaluate the generalizability of our findings. In addition, our analyses provide a *descriptive* characterization of the genetics of brain volumes in males and females. Differences in the number of genes mapped to genome-wide significant loci should be interpreted cautiously, as they may reflect variation in the strength of association signals rather than the presence of sex-specific loci. Despite these limitations, our study provides a complementary perspective to interaction-based models by illustrating how genetic association signals related to brain volumes are distributed across sexes. This approach offers useful neurobiological context while acknowledging that future studies using dedicated statistical frameworks will be required to formally quantify sex-differential genetic effects. Lastly, by identifying candidate loci and biological processes relevant to brain structure in females and males, our study highlights potential avenues for clarifying how genetic variation may contribute to sex variability in brain biology and disorders.

## Conclusion

In conclusion, while autosomal genetic influences on brain anatomy appear highly correlated between females and males, our gene-level findings suggest the possibility of sex-dependent features in the genetic underpinnings of brain structure. Further research is needed to explore sex-sensitive neurogenetics across different life stages, and in relation to the risk, presentation and course of mental and neurological disorders. Such evidence may improve our understanding of the relationship between sex, genetics and the brain, as well as their role in brain disorders. A better understanding of these relationships may also help identify developmental periods or life events during which genetic influences on the brain may shape risk differently in females and males.

## Supplementary Information

Below is the link to the electronic supplementary material.

Supplementary Material 1.

Supplementary Material 2.

Supplementary Material 3.

Supplementary Material 4.

Supplementary Material 5.

Supplementary Material 6.

Supplementary Material 7.

Supplementary Material 8.

## Data Availability

Data used in this study were obtained from the UK Biobank (28). GWAS summary statistics will be made openly available upon acceptance.

## Code availability

Codes and software needed to generate the results are available publicly.

MOSTest: https://github.com/precimed/mostest.

FUMA: https://fuma.ctglab.nl/.

LDSC: https://github.com/bulik/ldsc.
